# Isolated acute lupus pneumonitis as the initial presentation of systemic lupus erythematosus in an 8-year-old girl

**DOI:** 10.1007/s13317-018-0104-2

**Published:** 2018-03-27

**Authors:** Tuğba Şişmanlar Eyüboğlu, Ayşe Tana Aslan, Yeşim Özdemir, Deniz Gezgin Yıldırım, Necla Buyan, Öznur Boyunağa

**Affiliations:** 1 Department of Pediatric Pulmonology, Dr. Sami Ulus Maternity and Children Research and Training Hospital, Ankara, Turkey; 2 Department of Pediatric Pulmonology, Faculty of Medicine, Gazi University, Ankara, Turkey; 3 Department of Pediatric Nephrology, Faculty of Medicine, Gazi University, Ankara, Turkey; 4 Department of Pediatric Rheumatology, Faculty of Medicine, Gazi University, Ankara, Turkey; 5 Department of Radiology, Faculty of Medicine, Gazi University, Ankara, Turkey

**Keywords:** Acute lupus pneumonitis, Child, Hydroxychloroquine, Lung biopsy

## Abstract

Systemic lupus erythematosus (SLE) is a systemic autoimmune disease which has broad pleuropulmonary manifestations. One of the rare and mortal complications is acute lupus pneumonitis, which is reported very rarely, especially in childhood. Herein, we report an 8-year-old girl with isolated acute lupus pneumonitis as the initial presentation that required a lung biopsy for diagnosis. Although she had improvement with the administration of steroids, steroid treatment was reduced due to the drug’s side effects resulting in the addition of azathioprine and mycophenolate mofetil to the treatment regimen. After the new regimen failed to result in clinical improvement, hydroxychloroquine treatment was started and a significant improvement was observed. Acute lupus pneumonitis is an uncommon manifestation of SLE and diagnosis may be difficult in patients without other organ involvement.

## Introduction

Systemic lupus erythematosus (SLE) is a multisystem autoimmune connective tissue disease and commonly presents with arthritis, serositis, cutaneous manifestations, glomerulonephritis, and hematological and central nervous system involvement [1, 2]. Pulmonary involvement in SLE includes pulmonary hemorrhage, pulmonary hypertension, acute lupus pneumonitis, chronic interstitial pneumonitis, shrinking lung syndrome, pulmonary vasculitis, pulmonary embolism, bronchiolitis obliterans, cavitating pulmonary nodules, opportunistic pulmonary infections, and pleuritis, the most common [1, 3, 4]. Childhood onset SLE constitutes 20% of all lupus patients and pulmonary involvement has been reported in up to 80% of cases [5–8]. Acute lupus pneumonitis is rare in SLE and can be very difficult to diagnose in patients without any previous diagnosis of SLE. Herein, we report an 8-year-old girl with isolated acute lupus pneumonitis as the initial presentation.

## Case presentation

**Fig. 1:**
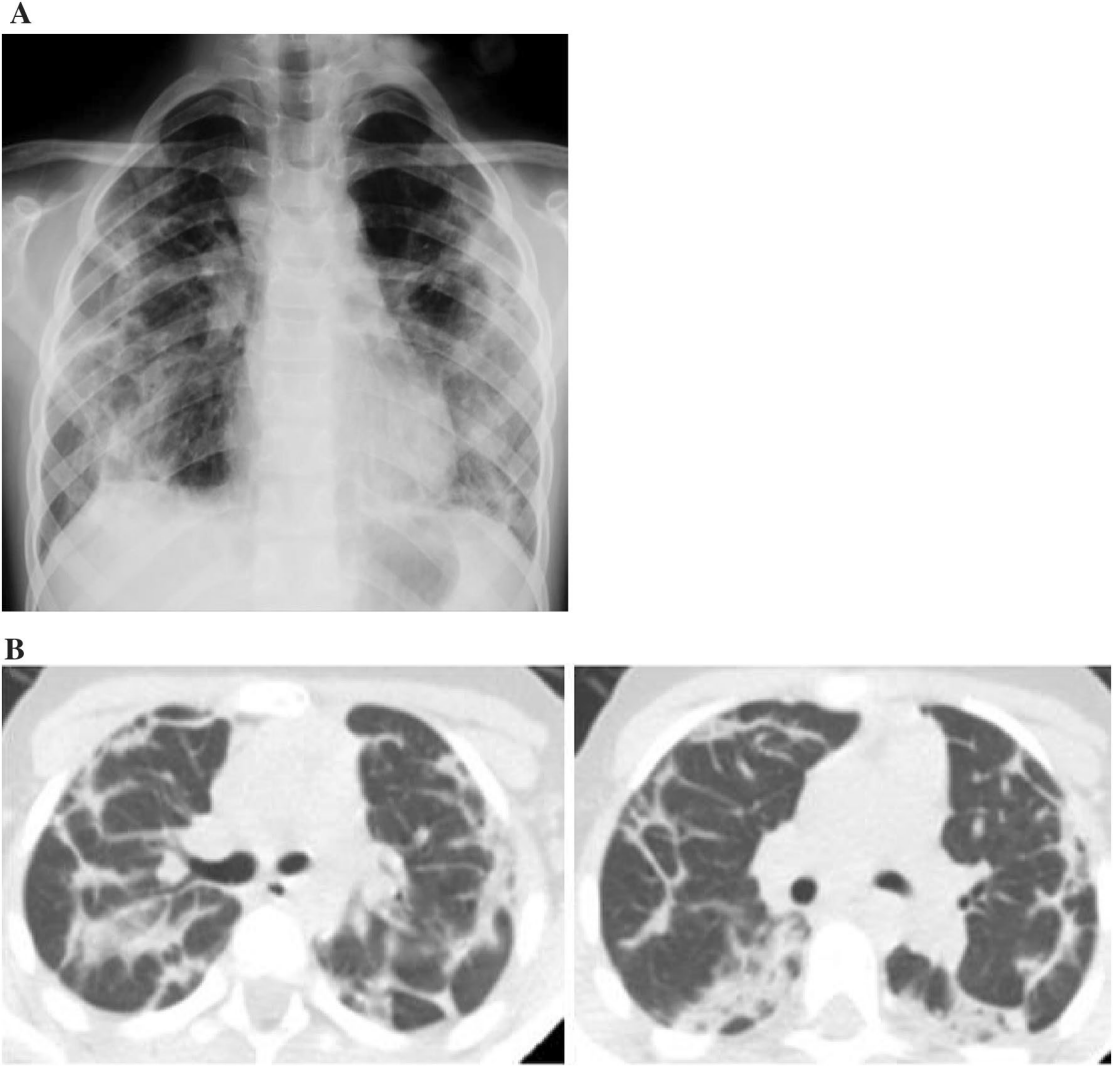
**a** Diffuse fibrotic changes and parenchymal consolidation on chest X-ray. **b** Common fibrotic changes, interlobular septal thickening, and subpleural parenchymal consolidation compatible with organising pneumonia on thorax CT

**Fig. 2:**
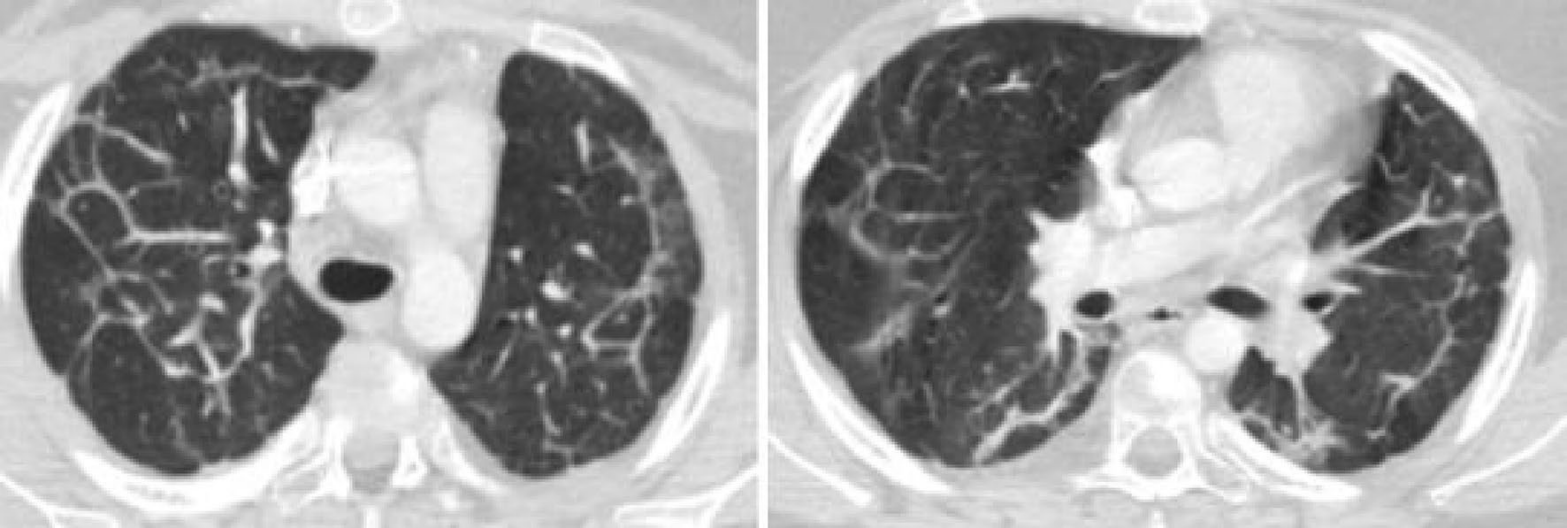
Fibrotic changes decreased with treatment on thorax CT

**Fig. 3:**
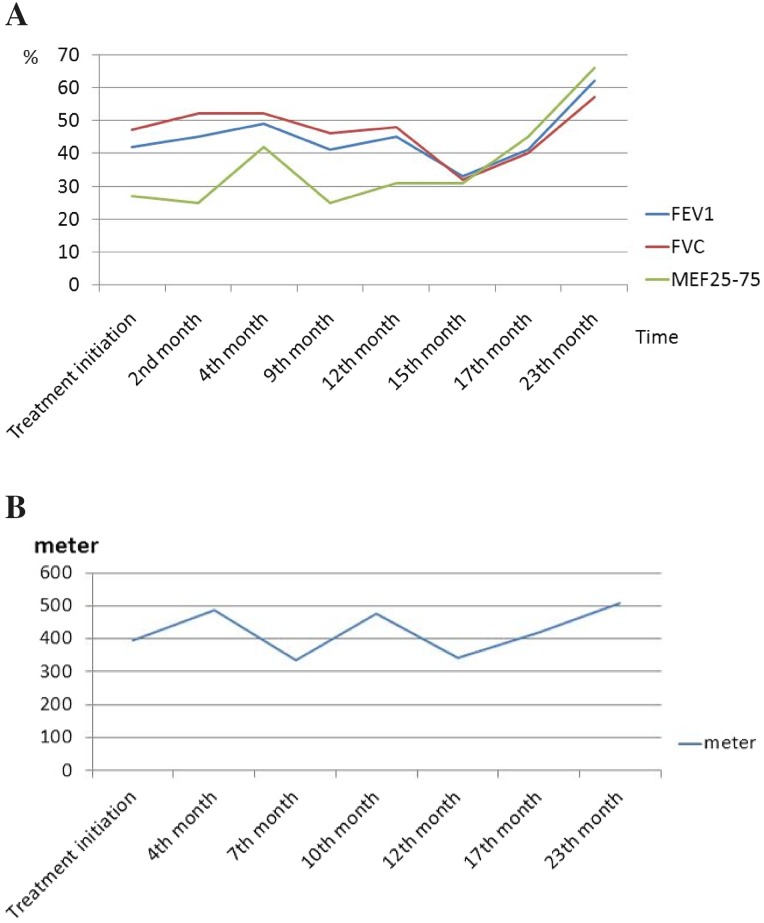
**a** Pulmonary function tests during the course of the disease. **b** 6-min walking test during the course of the disease

An 8-year-old female patient was referred to the pediatric pulmonology department with fever, dry cough, and weight loss for a duration of 1 month. She had been previously treated with intravenous antibiotic therapy and diagnosed with pneumonia at another hospital. Due to the lack of improvement despite standard treatment, she was referred for further investigation. She had no history of recurrent infection, exposure to birds or feathers, and reported no medication use. Physical examination revealed mild dyspnea and crepitant crackles on both lungs. There was no hypoxia. Diffuse fibrotic changes and parenchymal consolidation were present on chest X-ray. Thorax CT revealed that common fibrotic changes, interlobular septal thickening, and subpleural parenchymal consolidation were congruent with organising pneumonia (Fig. 1). Pulmonary function tests were compatible with restrictive pattern [forced expiratory volume in 1 s (FEV1): 0.70 L (42%), forced vital capacity (FVC): 0.94 L (47%), FEV1/FVC: 86%, forced expiratory flow (FEF) 25–75: 0.52 L/m (25%), all results are given as volume or flows and percentage of predicted]. Distance for the 6-min walk test was 394 m (52% of predicted). Her blood analysis showed hemoglobin of 13.8 g/dl, total white cell count of 7.08 × 10^3^/μL, and platelets at 277.5 × 10^3^/μL. Renal and liver function tests were within normal limits. Acute phase reactants were negative. All microbiological cultures including tuberculosis were negative. ANA++ and antiphospholipid antibodies IgM and repeated anti-dsDNA were positive [125–134 ıu/ml (normal: < 100)], whereas C3–C4 values were within normal limits. She had no proteinuria, hematuria, arthritis, rash, or hematological abnormalities. Her eye examination was normal. Echocardiography was normal without pulmonary hypertension. Bronchoscopy was performed and neutrophilic dominance was found in bronchoalveolar lavage (BAL); microbiological cultures were negative. Lung biopsy revealed that NSIP-like areas additionally showed inflammatory cell infiltration with superfluous plasma cells besides patchy consolidated areas with increased interstitial fibrosis and chronic pleuritis. She was diagnosed as acute lupus pneumonitis according to the SLICC 2012 criteria [9] and systemic steroid treatment was started. During the first 3 days, 15 mg/kg pulse steroid was given and continued with oral prednisolon (1 mg/kg/day). On the fourth month of treatment, although her ANA and anti-dsDNA were mostly negative, she had a cushingoid appearance, common stria on legs and back, osteoporosis (BMD − 4.2), and mild glaucoma. As a result, steroid treatment was tapered back and azathioprine and mycophenolate mofetil were started. After the steroid began being reduced, ANA and anti-dsDNA became positive once again. Because there was no improvement in pulmonary function tests and radiological findings after reducing steroid treatment, hydroxychloroquine treatment was added. Following addition of hydroxychloroquine to the treatment regimen, her radiological findings and pulmonary function tests were improved. Fibrotic changes decreased both on chest X-ray and thorax CT (Fig. 2); improvement of pulmonary function tests and 6-min walking distance are shown below (Fig. 3). To date, she is being followed continuously without complaints such as cough, dyspnea, and exercise intolerance for 2 years.

## Discussion

Acute lupus pneumonitis is a rare manifestation of SLE which is reported in 1–4% of SLE patients with a high mortality rate [4, 10]. Therefore, early diagnosis and treatment are essential in these patients. Our patient had many difficulties for an accurate diagnosis. She had only pulmonary involvement without any other systemic symptoms, resulting in an initial diagnosis of SLE. In this case, the major concern was preventing pulmonary infections. Infections are the main cause of death in SLE patients [11]. All microbiological cultures were negative including tuberculosis. The incidence of pulmonary tuberculosis is higher in SLE patients with a 6–15-fold higher risk than in the normal population [12–14]. BAL cultures were sterile and pulmonary infections were excluded. Viral pneumonias such as CMV were also excluded. Pulmonary hemorrhage was the other differential diagnose but was eventually excluded as she had no hemoptysis, anemia and was without hemosiderin-laden macrophages in BAL. Systemic vasculitis was another possibility but was excluded as there was no vasculitic rash, glomerulonephritis, or positive antibodies supporting vasculitis. Lung biopsy was required to fulfill the SLE criteria and is an invasive procedure in childhood.

High-dose steroids, azathioprine, cyclophosphamide, mycophenolate mofetil, and other immunosuppressive agents can be used in the treatment of acute lupus pneumonitis [4, 15, 16]. There are no clinical trials for this treatment and current treatment regimens are based solely on case reports and small investigative series [4]. Pulmonary function tests and radiological findings of our patient were improved and immunological markers became negative following steroid treatment. Due to the side effects of steroids, a reduced dosage was necessary, resulting in a deterioration of pulmonary functions and radiological findings concurrent with positive immunological markers. This was another finding that confirmed our diagnosis. Azathioprine and mycophenolate mofetil were added, but a significant improvement was observed only after the addition of hydroxychloroquine treatment. Hydroxychloroquine treatment is used for long-term protective effects against SLE-related organ damage; however, the percentage of SLE patients receiving hydroxychloroquine treatment was reported to be very low [16, 17]. Although hydroxychloroquine is not mentioned as a first-line therapy option, it can be use safely in acute lupus pneumonitis in children. We did not observe any side effects due to hydroxychloroquine treatment in this patient. Another explanation may be for failure of azathioprine and mycophenolate mofetil and success of hydroxychloroquine as hydroxychloroquine is a weak immunomodulator compared to other two agents or it was just delayed effect of azathioprine and mycophenolate mofetil which lead to success.

In the English literature, patients presenting with acute lupus pneumonitis as the initial presentation have other manifestations of SLE such as photosensitivity, alopecia, oral ulcers, malar rash, and glomerulonephritis [2, 4, 13]. Our patient had only pulmonary manifestation which made the diagnostic difficult and had to be diagnosed in accordance with other organ involvement.

In conclusion, interstitial lung involvement as the sole symptom in SLE is very rare, especially in childhood. Acute lupus pneumonitis is a rare and mortal manifestation of SLE that sometimes necessitates invasive procedures such as lung biopsy for a diagnosis. Hydroxychloroquine treatment can be used in children with acute lupus pneumonitis when steroid treatment is contraindicated due to side effects.

## References

[1] Keane MP, Lynch JP (2000). Pleuropulmonary manifestations of systemic lupus erythematosus. Thorax.

[2] Khor CG, Kan SL, Tan BE (2014). Pulmonary manifestation as initial presentation for systemic lupus erythematosus. Int J Rheum Dis.

[3] Aggarwal HK, Jain D, Mittal A, Rao A, Yadav RK, Jain P (2016). Systemic lupus erythematosus presenting as fulminant lupus pneumonitis: a rare case report. Reumatismo.

[4] Wan SA, Teh CL, Jobli AT (2016). Lupus pneumonitis as the initial presentation of systemic lupus erythematosus: case series from a single institution. Lupus.

[5] Veiga CS, Coutinho DS, Nakaie CM, Campos LM, Suzuki L, Cunha MT (2016). Subclinical pulmonary abnormalities in childhood-onset systemic lupus erythematosus patients. Lupus.

[6] de Jongste JC, Neijens HJ, Duiverman EJ, Bogaard JM, Kerrebijn KF (1986). Respiratory tract disease in systemic lupus erythematosus. Arch Dis Child.

[7] Delgado EA, Malleson PN, Pirie GE, Petty RE (1990). The pulmonary manifestations of childhood onset systemic lupus erythematosus. Semin Arthritis Rheum.

[8] Lilleby V, Aaløkken TM, Johansen B, Førre Ø (2006). Pulmonary involvement in patients with childhood-onset systemic lupus erythematosus. Clin Exp Rheumatol.

[9] Petri M, Orbai AM, Alarcón GS, Gordon C, Merrill JT, Fortin PR (2012). Derivation and validation of the Systemic Lupus International Collaborating Clinics classification criteria for systemic lupus erythematosus. Arthritis Rheum.

[10] Orens JB, Martinez FJ, Lynch JP (1994). Pleuropulmonary manifestations of systemic lupus erythematosus. Rheum Dis Clin North Am.

[11] Ciftçi E, Yalçinkaya F, Ince E, Ekim M, Ileri M, Orgerin Z (2004). Pulmonary involvement in childhood-onset systemic lupus erythematosus: a report of five cases. Rheumatology.

[12] Erdozain JG, Ruiz-Irastorza G, Egurbide MV, Martinez-Berriotxoa A, Aguirre C (2006). High risk of tuberculosis in systemic lupus erythematosus?. Lupus.

[13] Mok MY, Lo Y, Chan TM, Wong WS, Lau CS (2005). Tuberculosis in systemic lupus erythematosus in an endemic area and the role of isoniazid prophylaxis during corticosteroid therapy. J Rheumatol.

[14] Tam LS, Li EK, Wong SM, Szeto CC (2002). Risk factors and clinical features for tuberculosis among patients with systemic lupus erythematosus in Hong Kong. Scand J Rheumatol.

[15] Pego-Reigosa JM, Medeiros DA, Isenberg DA (2009). Respiratory manifestations of systemic lupus erythematosus: old and new concepts. Best Pract Res Clin Rheumatol.

[16] Lam NC, Ghetu MV, Bieniek ML (2016). Systemic lupus erythematosus: primary care approach to diagnosis and management. Am Fam Physician.

[17] Costedoat-Chalumeau N, Dunogué B, Morel N, Le Guern V, Guettrot-Imbert G (2014). Hydroxychloroquine: a multifaceted treatment in lupus. Presse Med.

